# Anatomy and surgery of pure neuroendoscopic infratentorial supracerebellar approach for resection of pineal region tumor

**DOI:** 10.1186/s41016-018-0127-6

**Published:** 2018-08-03

**Authors:** Yuanlong Zhang, Dezhi Kang, Changzhen Jiang, Xiaorong Yan, Zhangya Lin, Yuanxiang Lin, Xiyue Wu, Chenyang Wang

**Affiliations:** Department of Neurosurgery, The First Affiliated Hospital Of Fujian Medical University, Fuzhou, 350004 China

**Keywords:** Neuroendoscopy, Infratentorial supracerebellar approach, Surgery, Anatomy, Pineal region tumor

## Abstract

**Background:**

The pineal region tumors are surrounded by important structures. Neuroendoscopy has been increasingly used at home and abroad. This study is to simulate pure neuroendoscopic infratentorial supracerebellar approach for resection of pineal region tumor from the cadaveric head, and discuss the advantages and safety through this corridor.

**Methods:**

The anatomical structure for resection of pineal region tumor was visualized through pure neuroendoscopic infratentorial supracerebellar approach in three cadaveric heads. Three cases with pineal region tumors were retrospectively analyzed and summarized between June 2017 and December 2017. All cases were operated through pure neuroendoscopic infratentorial supracerebellar approach in the first affiliated hospital of Fujian medical university.

**Results:**

The anatomical structures of pineal region can be completely visualized by pure neuroendoscopic infratentorial supracerebellar corridor in the cadaveric head. Among the three cases, the first case was total resection, the second case was subtotal resection and the last case was partial resection. The postoperative pathology revealed cavernous hemangioma, germinoma and yolk sac tumor, respectively. The patients were followed-up for 1–6 months and had normal life.The KPS (karnofsky performance status) score was 100.

**Conclusion:**

The anatomical structure of the pineal region can be completely visualized and the tumor can be safely removed through pure neuroendoscopic infratentorial supracerebellar approach.

## Background

The pineal region tumors are surrounded by important blood vessels and nerves. The operation may be difficult because of long distance and poor light under microscope. Damage to the deep veins may lead to death [1]. Neuroendoscopy has been increasingly applied due to strong light, wide vision and less pull to surrounding brain tissues. Pure neuroendoscopic resection of pineal region tumor has been reported worldwide [2, 3]. More and more neurosurgeons have received this operation. To better understand the pineal region structure, we simulated the surgery of pure neuroendoscopic infratentorial supracerebellar corridor for resection of pineal region tumor from cadaveric head. Three cases with pineal region tumors were treated using neuroendoscopy, with positive results. Our study confirm the feasibility for the pineal region tumor resection by pure neuroendoscopic infratentorial supracerebellar approach.

## Materials and methods

### Materials

Pineal region of three cadaveric heads were fixed in formaldehyde and operated through neuroendoscopic infratentorial supracerebellar approach. Another three cases with pineal region tumors were retrospectively analyzed and summarized between June 2017 and December 2017 in the first affiliated hospital of Fujian medical university (Table 1). All cases were male, aged 26, 17 and 62 years, respectively. Preoperative symptoms included headache, dizziness, fatigue, diplopia and dullness. Preoperative MRI (magnetic resonance imaging) and CTA (computed tomography angiography) provided better understanding of the relationship between the tumor and peripheral structures. AFP (alpha-fetoprotein) and β-HCG (β-human chorionic gonadotropin) were also examined. The three patients had preoperative hydrocephalus. Two of them underwent endoscopic third ventriculostomy (ETV), while one received lateral ventricle drainage. However, the hydrocephalus persisted after removing the drainage tube and the patient subsequently underwent ETV.

**Table 1:** Three cases with pineal region tumors

NO.	Age (years)	Gender	Preoperative syndrome	Hydrocephalus management	Tumor size (cm)	Degree of resection	Pathology	Postoperative treatment	KPS
1	26	Male	Dizziness	ETV	2.6 × 2.5	Subtotal	Germinoma	Chemotherapy	100
2	17	Male	Headache,	ETV	2.5 × 1.8	Partial	Yolk sac	Radio/chemotherapy	100
3	62	Male	Fatigue	ETV	2.4 × 2.1	Total	Cavernous hemangioma	/	100

### Methods

**Fig. 1:**
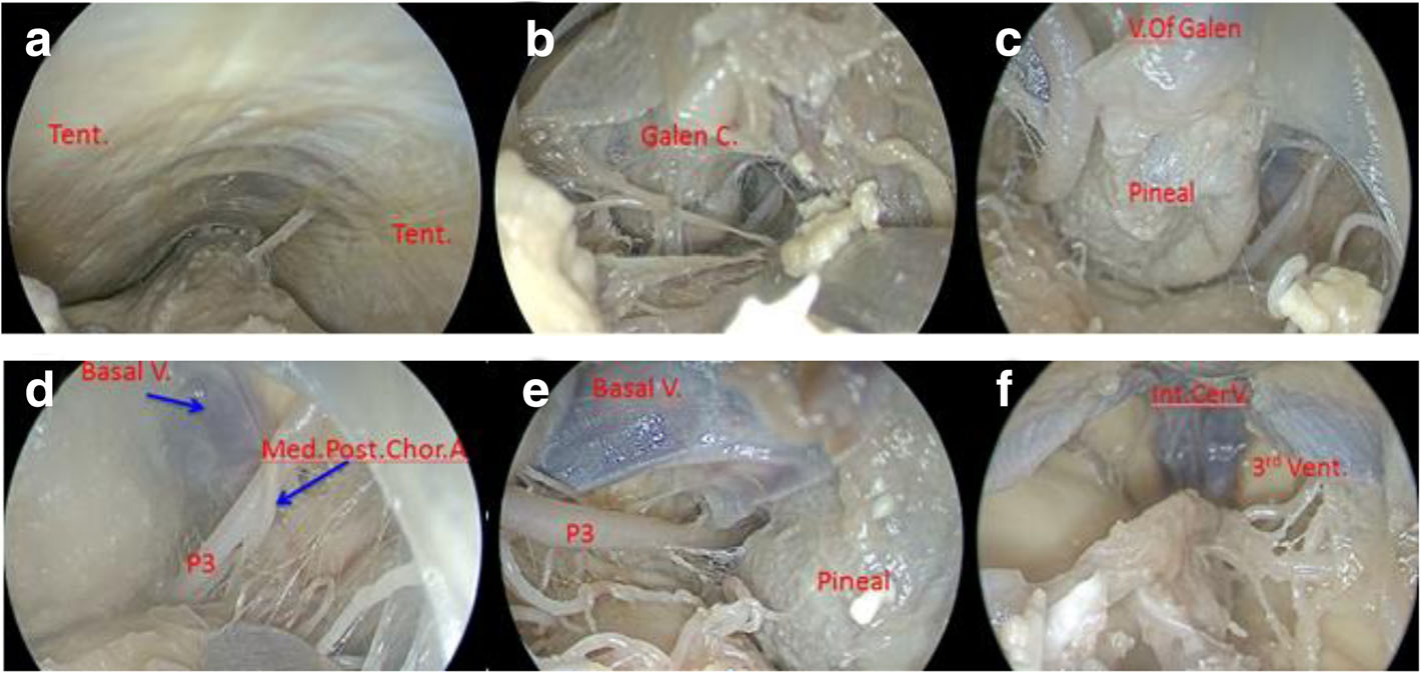
The anatomy of pineal region under neuroendoscopy: **a** Excising the superior cerebellar vermis vein, exposure of the quadrigeminal cistern; **b** Opening the thickened quadrigeminal cistern arachnoid; **c** Exposure of the pineal gland; **d** and **e** Peripheral structure of the pineal gland; **f** The third ventricle after removing the pineal gland

The cadaveric heads were fixed with a Mayfield holder, and skin incisions were made from inion to C3 vertebrae. The scalp, nuchal ligament and muscles were excised. Bone windows were exposed, with the upper bound to the confluence of sinuses and 1 cm above the proximal transverse sinus, and the lower bound to the upper edge of the foramen magnum. The dura was cut and suspended, then the bridging vein of the upper cerebellar vermis was clipped. Neuroendoscopy was used to visualize the anatomical structure of the pineal region to simulate the surgery from the middle gap under the tentorium, and the important anatomical structures were photographed in real time (Fig. 1).

**Fig. 2:**
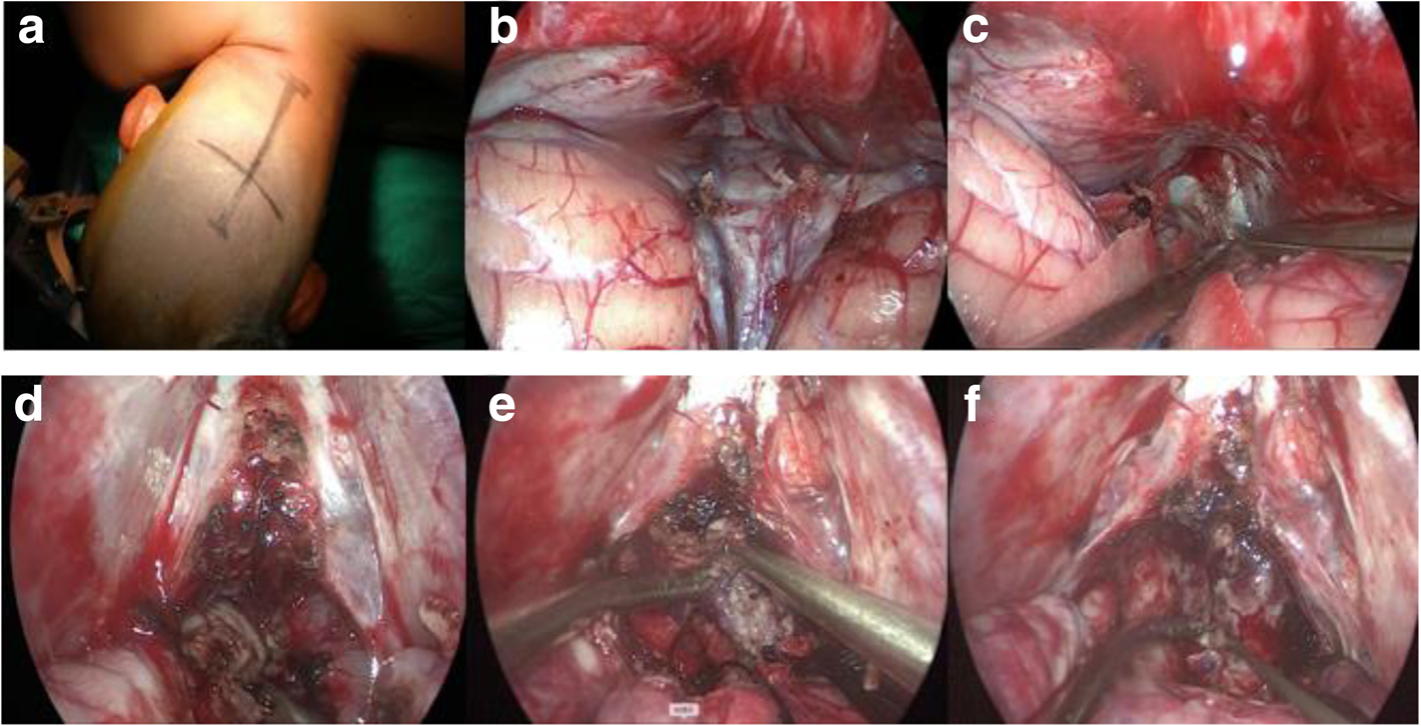
Resection of tumors in the pineal region under neuroendoscopy: **a** The lateral oblique position of the patient; **b** Exposure of the bridging vein of the upper cerebellar vermis; **c** Exposure of the quadrigeminal cistern; **d** Exposure of the tumor; **e** Resection of the tumor in pieces; **f** Exposure of the third ventricle after tumor removal

**Fig. 3:**
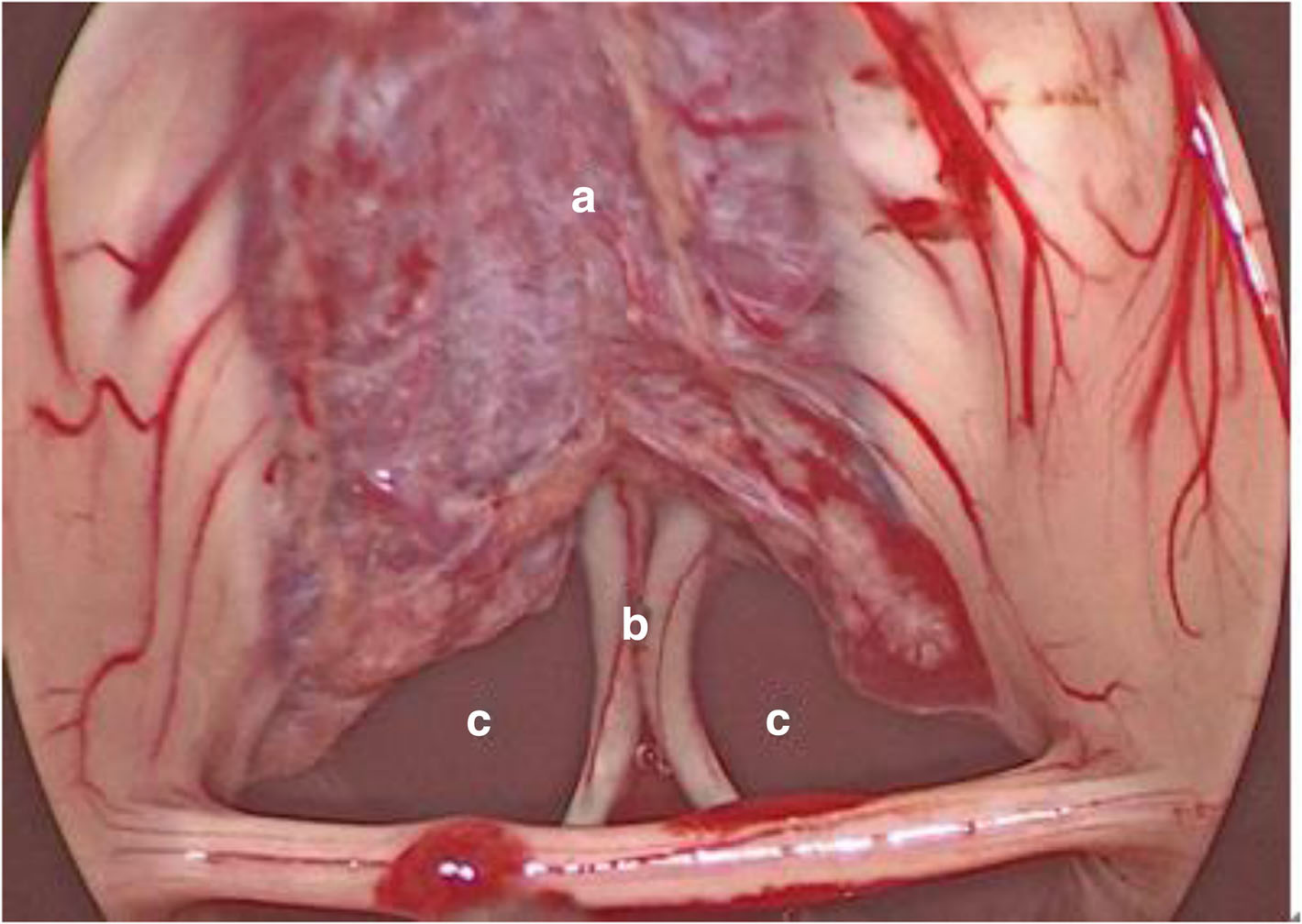
Exposure of the structures after tumor resection: internal cerebral veins (**a**), columella fornicis (**b**), Monro’s foramen (**c**)

The three patients were fixed in the lateral oblique position, with the upper body raised by 15 degrees and deviated to the right (Fig. 2a). The surgeries were conducted with suboccipital midline approach, from the inion to C1 vertebrae. A skull flap of 4 cm × 4 cm was opened, and the transverse sinus and torcular herophili were exposed. The neuroendoscope (4-mm diameter, 18-cm length, 0-degree lenses, Karl Storz, Germany) was introduced. The monitor was placed in front of the surgeon. The bipolar coagulation forceps and absorbable hemostat were used for performing intraoperative hemostasis.The cerebrospinal fluid was released from the cisterna magna for cerebellar retraction. The tumor was visible after excising the superior cerebellar vermis vein and separating the thickened quadrigeminal cistern arachnoid. In case 1, the tumor was tough and yellow, with usual blood supply. After sharp separation of basal vein arachnoid and the tumor, the neoplasm was resected in pieces to reduce the volume. Subtotal resection was conducted after intraoperative frozen section examination confirmed germinoma. In case 2, the tumor was crisp, yellow, with abundant blood supply. Obvious hemorrhage was observed after excising a small portion of the neoplasm. The intraoperative frozen section examination indicated yolk sac tumor, so partial resection was performed. In case 3, the tumor was soft and yellow. A coagulant clot was seen in the tumor after the cyst fluid was released. It was completely resected after removal of the capsule (Figs. 2b-f and 3). Postoperative pathological examination indicated cavernous hemangioma. After the operation, the dura was sewed with watertight manner. The bone flaps were recovered and the incisions were sutured. All three cases were treated with ETV before or after operation.

## Results

The anatomical structure of the pineal region can be fully visualized by pure neuroendoscopic infratentorial supracerebellar corridor. The vermis of the cerebellum and the great veins of the cerebrum can be observed from the middle gap under the tentorium. After excising the bridging veins between the tentorium and the superior surface of the cerebellum, the great cerebral veins were found to converge upward to the straight sinus, and the basilar and superior vermis veins and the bilateral internal cerebral veins converged into the large cerebral veins. The P3 segment of the posterior cerebral artery, the medial posterior choroidal arteries and the branch of the superior cerebellar artery were identified. The pineal gland was located under the bilateral internal cerebral veins.

**Fig. 4:**
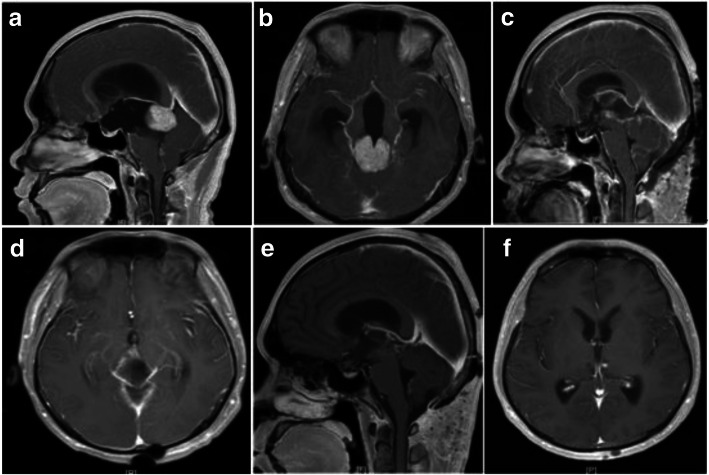
Preoperative and postoperative images of germinoma: **a** and **b** preoperative MRI shows tumor in the pineal region; **c** and **d** Three days after subtotal resection of the tumor; **e** and **f** Three months after operation, the tumor disappeared after radiotherapy and chemotherapy

**Fig. 5:**
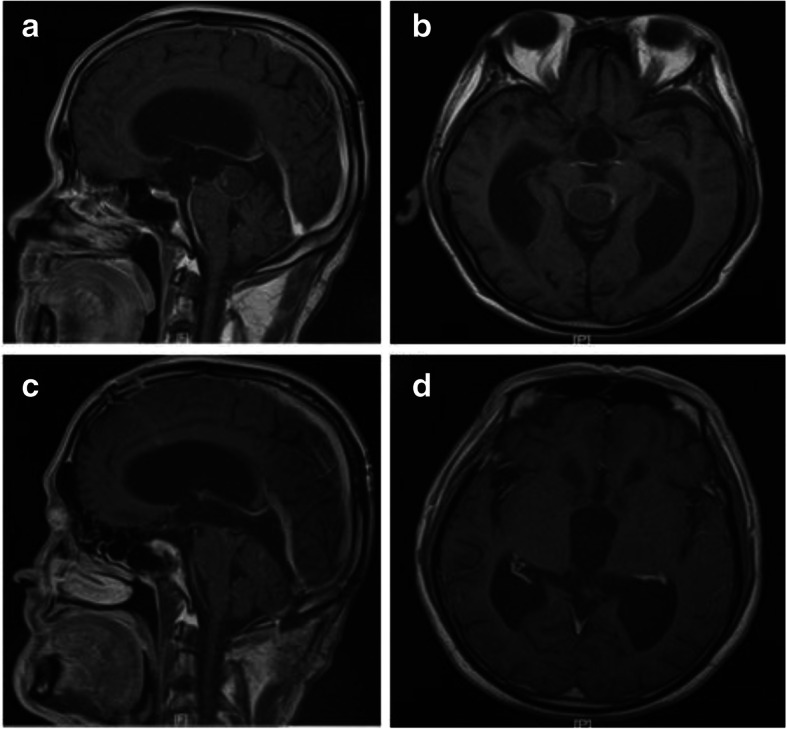
Preoperative and postoperative images of cavernous hemangioma: **a** and **b** preoperative MRI shows tumor in the pineal region; **c** and **d** One month after total resection of the tumor

The pineal region tumors were excised through the pure neuroendoscopic infratentorial supracerebellar corridor. The three cases underwent total resection, subtotal resection and partial resection, respectively. The results were positive at the early stage, and the KPS scores were 100. Postoperative pathological results of total tumor resection revealed cavernous hemangioma, subtotal resection revealed germinoma, and partial resection revealed yolk sac tumor. All the patients had obstructive hydrocephalus before operation. Two cases underwent ETV, while one received lateral ventricle drainage but suffered from headache after removing the drainage tube and subsequently underwent ETV. The preoperative symptoms of headache, dizziness, fatigue, diplopia and dullness were improved after operation. One case was treated with radiotherapy + chemotherapy, while another case was treated with radiotherapy. The follow-up duration was 1–6 months. All of them had normal life (Figs. 4 and 5).

## Discussion

Neuroendoscopy has the advantages of panoramic view, close observation, clear detail and ideal light. For hydrocephalus, brain tumor and arachnoid cyst, neuroendoscopy can replace the surgery under microscope to some extent [4]. Neuroendoscopy was commonly used in biopsy of the tumor tissue in the pineal region and ETV to treat hydrocephalus [5]. With the development of equipment and improvement of technology, neuroendoscopy is being increasingly used to resect the pineal region tumors, which requires the anatomy of the pineal region to be clearly visualized. The upper bound of the pineal region is the splenium of corpus callosum and interpositum, lower bound is the corpora quadrigemina and mesencephalic tectum, anterior border is the posterior part of the third ventricle, posterior boundary is the superior vermis. When simulating the infratentorial supracerebellar approach, the deep cerebral venous system, such as the great cerebral vein, the base vein, the superior vermis vein and the bilateral internal cerebral vein could be seen through the infratentorial gap. Reviewing the anatomical structure of the pineal region is useful to shorten the operation time and improve the quality of the surgery.

The approaches for pineal tumors include the occipital transtentorial corridor (Poppen approach), infratentorial supracerebellar corridor (Krause approach), posterior transcallosal interhemispheric approach and transcortical transventricular approach [6, 7]. The great cerebral vein and quadrigeminal venous rete can be clearly visualized from the Poppen corridor, but it may lead to occipital hemianopia. However, the tumor in front of the Galen vein cannot be seen. The advantage of the trans corpus callosum fornix approach is that it does not block the drainage vein, but may result in postoperative mutism and memory disorders. Cortex damage may be required in the lateral ventricle corridor, and epilepsy may be induced after operation. These approaches are often performed under a microscope. In order to expose the tumor, brain retractor is needed to pull the brain tissue, which may cause postoperative cerebellar edema.

The infratentorial supracerebellar corridor (Krause approach) was first used by Krause in 1926. This approach uses the natural passage between the cerebellum and tentorium to enter the pineal region, which can reduce damage to the surrounding tissues by using the space under the great cerebral vein [8]. It is suitable for tumors below the Galen vein complex. Majority of the tumors in the pineal region can be removed by this corridor [9]. When the tumor is resected under the microscope, the cerebellum is naturally sagged. There is a natural gap between the cerebellum and the tentorium, but the visual field is not fully exposed. Patients often need to sit during the operation, which is likely to cause gas embolism and other complications. Neuroendoscopy can provide sufficient surgical field. The patient can remain in prone position during the operation. It is not necessary to pull the brain to expose the tumor, which reduces postoperative cerebellar edema and other injuries.

Tumors of the pineal region account for 3–8% of all central nervous system tumors [10]. The pathological types include germinoma, nongerminomatous germ cell tumors (teratoma, embryonal carcinoma, choriocarcinoma), pineal parenchymal tumors (pineocytoma and pineoblastoma), glial tumor (ependymoma) and other tumors (cavernous hemangioma and meningiomas). The tumor is deep in the brain, surrounded by important blood vessels and nerve tissues. Hence, the surgery is very risky and difficult. The patient usually also has hydrocephalus, which further increases the complexity of surgery. Surgical resection remains the most important method for most pineal tumors, except germinoma [11]. The latter can be treated with radiotherapy and chemotherapy after a clear diagnosis by tumor biopsy. Stereotactic biopsy of the pineal tumor has been conducted in some cases, but it usually causes complications, such as postoperative bleeding [12].

About 60% of all pineal region tumors are germinomas [13]. Germinoma is mostly found in young patients, under 25 years of age [14]. In the first case, preoperative biochemistry and magnetic resonance examination indicated germinoma, which was confirmed by intraoperative frozen section examination. So subtotal resection was performed for protecting the surrounding deep structure. The surgery was safe, and the damage caused by postoperative radiotherapy and chemotherapy was minimized. The patient recovered well.

Yolk sac is a malignant nongerminomatous germ cell tumor, and its incidence rate is higher in the pineal region than in the saddle area [15]. In the second case, the tumor was partially resected due to abundant blood supply and hemostatic difficulties. Postoperative treatment included radiotherapy and chemotherapy. The patient recovered well. Biopsy is preferred under the neuroendoscope through the infratentorial supracerebellar corridor because it does not cause tumor spread and metastasis. Bleeding is rare and does not cause obstructive hydrocephalus or destroy the deep structure. The specimens can be obtained under direct vision, and the operation is completed after frozen pathological confirmation.

The cavernous hemangioma is a cavernous vascular malformation, which is a type of cerebral vascular malformation, and its incidence is 0.9% [16]. In the third case, preoperative MRI and CTA suggested benign lesions. Total resection was achieved by neuroendoscopy, without damage to the brain stem. The postoperative follow-up indicated good recovery. Therefore, preoperative MRI and CTA imaging examinations are essential to diagnose benign and malignant lesions. Combined with intraoperative frozen pathology for the diagnosis of cavernous hemangioma, the total resection of tumor can be achieved.

Neuroendoscopy is not suitable for all pineal tumors. It should be used carefully for tumors which are tough, obviously calcified, with abundant blood supply and surrounded by arteries. The tough and calcified tumor is difficult to remove by neuroendoscopy, which increases the difficulty of operation. Profuse bleeding occurs under neuroendoscopy in hypervascular tumors, which increases the surgical risk. The tumor is best confined to the quadrigeminal cistern, not beyond the splenium of corpus callosum and under the Galenic complex, both sides not exceeding the pulvinar [2] because it is safe and feasible to resect the neoplasm under neuroendoscopy. It was difficult to surpass both sides of P3 and Galen’s veins because of the limitation of anatomical structure in our simulated neuroendoscopic operation.

In summary, the anatomical structure of the pineal region can be completely visualized with enough space for surgery through pure neuroendoscopic infratentorial supracerebellar corridor. Preoperative comprehensive MRI and CTA examinations are crucial for complete neuroendoscopic surgery.

## Abbreviations

AFP: alpha-fetoprotein
CTA: computed tomography angiography
ETV: endoscopic third ventriculostomy
KPS: karnofsky performance status
MRI: magnetic resonance imaging
β-HCG: β-human chorionic gonadotropin

## References

[1] Matsutani M, Sano K, Takakura K, et al. Primary intracranial germ cell tumors: a clinical analysis of 153 histologically verified cases. J Neurosurg. 1997;86(3):446–55. 10.3171/jns.1997.86.3.0446. 10.3171/jns.1997.86.3.0446 9046301

[2] Gu Y, Hu F, Zhang X. Purely endoscopic resection of pineal region tumors using infratentorial supracerebellar approach: How I do it. Acta Neurochir (wien). 2016;158(11):2155–8. 10.1007/s00701-016-2895-0. 10.1007/s00701-016-2895-0 27506850

[3] Sood S, Hoeprich M, Ham SD. Pure endoscopic removal of pineal region tumors. Child’s Nerv Syst. 2011;27:1489–92. 10.1007/s00381-011-1490-1. 10.1007/s00381-011-1490-1 21607639

[4] Derakhshan N, Masoudi MS. Transient mutism following neuroendoscopy in children; report of two cases. Chinese Neurosurg J. 2016;2(20). 10.1186/s41016-016-0038-3.

[5] Ellenbogen RG, Moores LE. Endoscopic management of a pineal and suprasellar germinoma with associated hydrocephalus: technical case report. Minim Invasive Neurosurg. 1997;40:13–5. 10.1055/s-2008-1053406. 10.1055/s-2008-1053406 9138302

[6] Kolovalov AN, Pitskhehnri DI. Principles of treatment of the pineal region tumors. Surg Neurol. 2003;59(4):250–68. 10.1016/S0090-3019(03)00080-6. 10.1016/s0090-3019(03)00080-6 12748006

[7] Azab WA, Nasim K, Salaheddin W. An overview of the current surgical options for pineal region tumors. Surg Neurol Int. 2014;5:39. 10.4103/2152-7806.129430. 10.4103/2152-7806.129430 PMC4014815 24818046

[8] Hernesniemi J, Romani R, Albayrak BS, et al. Microsurgical management of pineal region lesions: personal experience with 119 patients. Surg Neurol. 2008;70:576–83. 10.1016/j.surneu.2008.07.019. 10.1016/j.surneu.2008.07.019 19055952

[9] Hart MG, Santarius T, Kirollos Rw. How I do it—pineal surgery: supracerebellar infratentorial versus occipital transtentorial. Acta Neurochir. 2013;155(3):463–7. 10.1007/s00701-012-1589-5. 10.1007/s00701-012-1589-5 23269352

[10] Abay EOII, Laws ER Jr, Grado GL, et al. Pineal tumors in children and adolescents. Treatment by CSF shunting and radiotherapy. J Neurosurg. 1981;55:889–95. 10.3171/jns.1981.55.6.0889. 10.3171/jns.1981.55.6.0889 7299463

[11] Radovanovic I, Dizdarevic K, de Tribolet N, Masic T, Muminagic S. Pineal region tumors-neurosurgical review. Med Arh. 2009;63:171–3. PMID: 20088167 20088167

[12] Quick-Weller J, Lescher S, Baumgarten P, et al. Stereotactic biopsy of pineal lesions. World Neurosurg. 2016;96:124–8. 10.1016/j.wneu.2016.04.130. 10.1016/j.wneu.2016.04.130 27287513

[13] Al-Hussaini M, Sultan I, Abuirmileh N, et al. Pineal gland tumors: experience from the SEER database. J Neuro-Oncol. 2009;94(3):351–8. 10.1007/s11060-009-9881-9. 10.1007/s11060-009-9881-9 PMC2804886 19373436

[14] Villano JL, Propp JM, Porter KR, et al. Malignant pineal germ-cell tumors: an analysis of cases from three tumor registries. Neuro-Oncology. 2008;10(2):121–30. 10.1215/15228517-2007-054. 10.1215/15228517-2007-054 PMC2613814 18287340

[15] Sawamura Y, Ikeda J, Shirato H, et al. Germ cell tumours of the central nervous system: treatment consideration based on 111 cases and their long-term clinical outcomes. Eur J Cancer. 1998;34(1):104–10. PMID: 9624246. 10.1016/s0959-8049(97)10045-4 9624246

[16] Berk C, Shaya M, Acharya R, et al. Surgical management of intracrnial cavenous malformations: the Louisiana State University health sciences center, Shreveport experience. South Med J. 2005;98(6):611–5. PMID: 16004168. 10.1097/00007611-200506000-00007 16004168

